# Assessment of Dietary Intake of Schoolchildren Living in Urban Settings: A Case Study of Karaganda City

**DOI:** 10.3390/nu18101507

**Published:** 2026-05-08

**Authors:** Svetlana Rogova, Olga Plotnikova, Karina Nukeshtayeva, Zhanerke Bolatova, Olzhas Zhamantayev, Aza Galayeva, Denis Turchaninov

**Affiliations:** 1 School of Public Health, Karaganda Medical University, Karaganda 100008, Kazakhstan; s.rogova@qmu.kz (S.R.); bolatovazhanerke93@gmail.com (Z.B.); 2 Department of Occupational Hygiene and Occupational Pathology, Omsk State Medical University, Omsk 644099, Russia; olga.plotnikova7@mail.ru; 3 Department of Hygiene and Human Nutrition, Omsk State Medical University, Omsk 644099, Russia; omskgsen@yandex.ru

**Keywords:** nutrients, vitamins, actual nutrition, Kazakhstan, children, urban setting

## Abstract

**Background/Objectives**: Kazakhstan currently lacks representative data on the actual consumption of calories, macronutrients, and micronutrients among students, despite the rising interest in the subject of children’s nutrition. The objective of the study was to assess the actual nutritional status of urban schoolchildren and identify differences depending on demographic and socioeconomic factors. **Methods**: A 24 h dietary survey was used. The final analytical dataset included 865 children aged 7 to 17 years, representing only the two specified ethnocultural groups. We divided these participants into three groups according to standard age classification: 7 to 10 years, 11 to 14 years, and 15 to 17 years. We calculated the nutrient profile using national and international food composition databases. **Results**: We found an age-related trend toward increased consumption of fluids, proteins, fats, and several micronutrients. Adolescents (15–17 years) had a higher intake of simple sugars and a lower intake of starch compared to other age groups. Age, gender, ethnicity, and socioeconomic factors (family income and food expenditure) were statistically significantly associated with BMI and diet composition, with caloric intake and essential nutrient intake increasing with age. **Conclusions**: The results emphasize the need for comprehensive interventions that go beyond school meals, such as controlling the food environment, lowering the availability of ultra-processed foods, boosting the availability of dairy products, fruits, and vegetables, and creating mechanisms for tracking and assessing the efficacy of Kazakhstan’s revised school meal standards.

## 1. Introduction

The relevance of the organization and quality of nutrition for schoolchildren is due to its significant impact on the growth, development, cognitive functions and prevention of chronic diseases of the younger generation [1,2]. Currently, there are both micronutrient deficiencies, including anemia and stunting, and an increase in the prevalence of overweight and obesity among school-aged children [3,4]. The shift from healthy foods to energy-dense, processed, and micronutrient-poor foods, as well as failure to maintain energy balance, is associated with the risk of both malnutrition and overweight and obesity [1,5].

An optimal protein, fat, and carbohydrate (PFC) ratio is key to ensuring children’s normal physical, cognitive, and social development. According to international recommendations, protein in schoolchildren’s diets should account for 10–15% of total calories, fat for 25–35%, and carbohydrates for 50–60% [2,3]. However, as modern research shows, children often have an imbalance in the macronutrient composition of their diet, including due to excessive consumption of saturated fats and free sugars [6,7].

The micronutrient composition of schoolchildren’s diets is just as important as the macronutrients. Vitamin and mineral deficiencies are widespread among school-age children, as confirmed by international studies. For example, in a study of Lebanese schoolchildren, vitamin A deficiency was found in 69.5% of children [2]. Similar trends have been identified in European countries, where a significant proportion of children do not achieve recommended levels of iron and folate intake [8,9].

Micronutrient deficiencies in children lead to complex and often irreversible impairments in physical, cognitive, and emotional development, as confirmed by modern research. They are associated with impaired growth and bone formation, muscle weakness, as well as delayed motor development and decreased hand–eye coordination. Furthermore, micronutrient deficiencies are associated with impaired neural connections and decreased executive functions, memory, and attention, as well as impaired intellectual development and academic performance [10,11,12]. Iron and B vitamin deficiencies in early life increase the risk of emotional and behavioral problems, decreased social maturity, and impaired emotional regulation [12,13,14].

The Karaganda agglomeration is in the central part of the Republic of Kazakhstan and is a large industrial and urbanized region. The agglomeration’s population as of 2024 was approximately 525,000 people. The region’s socioeconomic indicators are characterized by a moderate unemployment rate (approximately 4.8%) and stable income levels (the average monthly salary is approximately 976$), reflecting the industrial-oriented structure of the economy. The region’s ethnic composition is multiethnic, based on national census data. Kazakhs and other ethnic groups predominate in the population structure [15]. In Kazakhstan, where urbanization and dietary transition are ongoing, these processes are particularly important for assessing the actual nutritional status of schoolchildren. Available data indicate a shift in dietary patterns toward higher consumption of fats, added sugars, and ultra-processed foods [16,17,18]. This contributes to a double burden of nutrition, in which micronutrient deficiencies are combined with rising prevalence of overweight and obesity. In large cities in Kazakhstan, the impact of these processes may be exacerbated by the heterogeneity of the urban food environment, including differences in the availability of food outlets, fast food outlets, and retail outlets offering foods with varying nutritional values [19,20,21]. As a result, schoolchildren living in different areas of the same city may experience different conditions when it comes to choosing food and forming their daily diet.

Despite growing interest in this issue, Kazakhstan remains short of representative data on the actual consumption of energy, macro-, and micronutrients among urban schoolchildren. Available studies were either conducted before the nutritional standards were updated [22,23,24] or were limited to anthropometric indicators without assessing the chemical composition of the diet [25,26]. Furthermore, differences in dietary patterns related to gender, ethnicity, and residential area within the city remain understudied. This gap limits the ability to more accurately assess nutritional risks in urban school populations and complicates the comparison of data from Kazakhstan with research results from other countries also experiencing urbanization and changing food environments.

Additionally, 24 h dietary recall is a widely used method for assessing dietary intake in population-based studies and allows characterization of energy, macro- and micronutrient intake in schoolchildren, as well as assessment of dietary compliance with current recommendations.

The aim of the study was to assess the actual nutritional status of urban schoolchildren and identify differences depending on age, gender, ethnicity, and region of residence.

## 2. Materials and Methods

### 2.1. Study Design and Sampling Strategy

We conducted a descriptive cross-sectional study using a purposive two-stage sampling strategy among schoolchildren aged 7 to 17 years in public schools in Karaganda. Data collection took place between September 2025 and February 2026. The research protocol involved no dietary interventions. We recorded actual food consumption during the 24 h preceding the interview to perform a comparative analysis of daily energy and nutrient intake across age groups (7 to 10, 11 to 14, and 15 to 17 years), genders, residential districts (“Maikuduk” and “South-East”), and ethnocultural backgrounds. Each participant completed a single 24 h recall.

We targeted six public schools per district, including 12 schools in total. The city education department distributed initial invitations to all schools in these areas. School administrations supported the study by informing parents, providing interview facilities, and distributing research materials. We recruited participants through consecutive enrollment of eligible schoolchildren on survey days, aiming for a proportional distribution of approximately 69 to 70 schoolchildren per school.

### 2.2. Ethical Considerations and Inclusion/Exclusion Criteria

Participation required written informed consent from parents or legal guardians for children up to 17 years old, along with voluntary written assent from adolescents aged 15 to 17 years. Researchers briefed respondents on the study objectives and data collection procedures before the interviews began. The Karaganda Medical University Ethics Committee approved the study protocol (Protocol No. 16, 16 September 2025). The research adhered to the ethical principles of the Declaration of Helsinki. We anonymized data by assigning unique codes to participants, storing the matching key separately, and restricting database access strictly to the core research team.

Children entered the study if they attended a selected public school, met the age requirements, possessed signed consent forms, and lived permanently in the city without extended trips to rural areas during the week before the interview. We excluded participants based on parent-reported chronic conditions requiring therapeutic diets, acute febrile illnesses on or right before the interview day and diagnosed developmental or behavioral disorders interfering with participation. We also excluded cases with incomplete dietary questionnaires, unresolvable data entry errors, and voluntary withdrawals. Quality control procedures flagged implausible energy values and portion sizes. Researchers attempted to verify these anomalies through follow-up contact and excluded the data if verification failed.

We collected data exclusively during the academic season. Schoolchildren establish stable daily routines after summer vacations, allowing accurate assessment of diets alongside school meals. We scheduled interviews only for school days, actively excluding weekends, holidays, and school break periods.

### 2.3. Sample Size Calculation

We calculated the minimum sample size using Cochran’s formula for a simple random sample:

n_0_ = (Z^2^ × p × (1 − p))/e^2^

Using a 95% confidence level (Z = 1.96), maximum variability (p = 0.5), and a 5% margin of error (e = 0.05), the baseline calculation yielded n_0_ = 384.

Applying a finite population correction for the estimated 50,000 schoolchildren in Karaganda, the adjusted minimum sample size became *n* = 381. To account for the cluster sampling design at the school level, we applied a design effect factor of DEFF = 2.0, reflecting typical intra-cluster correlation values in school-based nutrition surveys [27]. This step produced a cluster-adjusted sample size of n_cluster = 762. Anticipating a 10% non-response rate, we expanded the target sample to 838 students.

Parents reported ethnocultural background for younger children, while adolescents self-identified. For the comparative analysis, we grouped participants into two broad categories: Kazakhs and non-Kazakhs (Russians, Ukrainians, Belarusians, and others). The final analytical dataset comprised 865 children aged 7 to 17 years, exclusively representing the two specified ethnocultural groups. We stratified these participants according to standard age periodization into three groups: 7 to 10 years, 11 to 14 years, and 15 to 17 years. Age stratification (7–10, 11–14, and 15–17 years) distinguishes between early, middle, and late adolescence. This division corresponds to stages of learning and psychophysiological development, separating prepuberty and adolescence, which is necessary for interpreting age-related differences in nutrition.

### 2.4. Dietary Assessment and Data Processing

We employed the 24 h dietary recall method [28,29] through a multi-stage interview process. The steps included listing all consumed foods and beverages, detailing dish composition and cooking methods, and confirming portion sizes using a standardized photographic atlas [30], food models, and household measures. A final step involved checking the record for logical consistency. Interviewers conducted the sessions in either Russian or Kazakh. For children aged 7 to 10 years, parents or guardians provided the dietary data. To quantify school meals for this younger group, trained teachers estimated the consumed fraction of the standard school portion using a standardized scale (1.0, 0.5, 0.25, 0). For adolescents aged 11 to 17 years, participants self-reported their intake.

We calculated the nutrient profile using national food composition databases [30,31,32,33,34,35]. For complex dishes, our team applied standardized recipes, selecting analogs that closely matched the dish name, ingredients, and cooking method. The final dataset for each participant integrated sociodemographic characteristics, anthropometric measurements, and dietary intake. The sociodemographic module captured age, gender, ethnicity, residential district, school shift, family income category, and household food expenditures (in the Karaganda region, according to the Bureau of National Statistics of the Republic of Kazakhstan, the average monthly salary in 2025 was about $976; the median salary in the Republic of Kazakhstan is approximately $731; the minimum wage is set at $183). Unspecified income and food expenses were coded as separate categories and included in the regression along with known values.

We measured body weight and height at school using medical scales and stadiometers to calculate body mass index (kg/m^2^) and classify nutritional status. The distribution of participants by nutritional status categories, calculated based on BMI-for-age z-scores (WHO), is presented in the Section 3. Thresholds according to the WHO: “underweight”: z < –2, “normal weight”: –2 ≤ z ≤ +1, “overweight”: +1 < z ≤ +2, and obesity: z > +2.

Dietary metrics derived from the recalls included total daily energy intake (kcal/day) and the daily consumption of proteins, fats, carbohydrates, and dietary fiber (g/day). We quantified micronutrients such as sodium, potassium, calcium, magnesium, phosphorus, iron, and cholesterol (mg/day), vitamin A (mcg/day), vitamin E (mg/day), vitamins B1, B2, C, and PP (mg/day). When available in the databases, we analyzed the fatty acid profile. The 24 h recall method allowed for the recording of all food, meals, and beverage consumption, regardless of where it was consumed. The “fluid” indicator reflected total water intake and included water and beverages (including sugary soft drinks), liquid dishes (soups), and water contained in food products. Sugars from beverages were included in the calculation of mono- and disaccharides and were included in the total carbohydrate indicator.

### 2.5. Statistical Analysis

Descriptive statistics were used to summarize categorical variables as frequencies and percentages, and continuous variables as means and standard deviations. Each model was constructed using several independent variables related to socioeconomic status, gender, age, and ethnicity. Model selection was based on theoretical significance and empirical correlation, and all predictor variables were included in the regression analysis simultaneously. Multicollinearity was assessed using the tolerance statistic and the variance inflation factor (VIF), ensuring that all included variables met acceptable thresholds (T > 0.1; VIF < 4). Separate models were developed for each nutrient.

## 3. Results

### 3.1. Characteristics of the Sample

Table 1 presents the characteristics of the sample of 865 schoolchildren aged 7–17 years, divided into three age groups: 7–10 years (*n* = 288), 11–14 years (*n* = 309), and 15–17 years (*n* = 268). By gender, the overall sample was almost evenly distributed: 414 boys (47.86%) and 451 girls (52.14%). In terms of ethnicity, most of the participants were children of Kazakh ethnicity—478 (55.26%). Most participants (53.87%) attended schools in the “South-East” district. A total of 71.56% schoolchildren had classes in the morning (morning shift).

An analysis of family income revealed that 32.60% of families had an income of less than $950 per month, while 22.31% had an income of more than $1200 per month.

A relatively equal number of respondents spend less than $310, $310–430, and more than $430 on food per month. In terms of mothers’ education level, the majority had higher education—675 (78.03%). Of the fathers, 510 (58.96%) had higher education.

The distribution of BMI-for-age categories shows that the majority of children and adolescents were of normal weight (mean z-score = −0.30 ± 0.62, min = −1.96, max = 0.99), while overweight (overweight (mean z-score = 1.43 ± 0.30, min = 1.00, max = 1.94) and obesity (2.39 ± 0.29, min = 2.02, max = 3.23)) accounted for a significant 15.14% of the sample. Girls had a higher prevalence of overweight compared to boys, while obesity rates were relatively comparable between the genders (Table 2).

Differences by gender and age are revealed. In the younger age group (7–10 years), boys are relatively more likely to be obese than girls. In the 11–14 age group, there is a shift toward a higher prevalence of overweight in girls, while obesity rates in this group remain relatively moderate. In the older age group (15–17 years), the trend toward a higher proportion of overweight among girls persists, while this category is less common among boys. However, no statistically significant difference in z-score values by gender was found for each age category.

Underweight is extremely rare and does not show a clear relationship with either age or gender.

### 3.2. Actual Nutrition per Day of Schoolchildren Depending on Age and Weight Category

In the younger age group (7–10 years), the nutritional profile is characterized by a relatively balanced intake of macronutrients, but with several peculiarities. The average daily calorie intake in this group is 2132.26 kcal (sd = 369.99) and corresponds to the recommended energy intake (2100 kcal). The diet of children of this age is characterized by their consumption of carbohydrates below the recommended norm (287.02 ± 64.32 out of 315 g recommended per day, 91%), mainly due to starch (161.74 ± 60.48 g). At the same time, the consumption of simple sugars remains moderate compared to older age groups. Regarding the consumption of minerals, children in the younger age group consume about 2.5 g of sodium and 2.3 g of potassium, which exceeds the recommended norms by almost two times (recommended norms: 1.2 g for sodium and 0.90 g for potassium). Table 3 shows that the average calcium intake in this group is 587.59 ± 181.71 mg, which is almost 2 times less than the recommended norm for this age (the recommended calcium intake for children 7–10 years is 1000 mg). The intake of other minerals remains within the recommended norms. Regarding vitamin intake, a deviation is observed in the daily intake of vitamin C: 83.18 ± 60.66 mg with 45 mg recommended (Table 3).

An analysis of the diet of children aged 7–10 years, depending on their weight category, revealed that protein consumption in all weight categories corresponds to the exceeded norm of 63 g per day. The highest protein consumption is observed in the underweight group of children (74.64 g) and in the normal weight group (73.31 g). In all weight categories, an underconsumption of the recommended carbohydrate intake is noted, and the highest carbohydrate consumption is observed in children with normal weight (289.35 ± 72.99 g). Excessive fat consumption is noted in all weight categories, especially in the overweight and obese groups (82.24 and 77.62 g, respectively). Daily energy consumption in all groups is slightly different from the recommended norms (2100 Kcal), both up and down, from 2060.33 calories in underweight children to 2143.61 calories in obese children.

In the middle age group (11–14 years), the nutritional profile is characterized by relative variability and signs of a less balanced diet compared to younger children. The average daily energy intake in this group is 2109.52 kcal (sd = 398.32), which generally does not meet the recommended standards for both boys and girls (the recommended norm for boys is 2500 kcal, for girls 2400 kcal). The diet of adolescents in this group is also distinguished by a reduced carbohydrate intake compared to the recommended values (251.53 ± 64.84 at 375 g for boys and 360 g for girls per day), which is largely associated with a decrease in the proportion of starch (130.09 ± 44.07 g). At the same time, a lower consumption of simple sugars is noted compared to other age groups. Against this background, there is an increase in fat consumption (87.79 ± 26.09 g), including saturated fatty acids, which indicates a shift in the diet structure towards higher fat foods and exceeds the recommended norm by 13% (75–75 g recommended norm). Protein consumption remains relatively stable and does not demonstrate significant deviations from the recommended norms (72–75 g). Analysis of mineral intake shows that the sodium level remains high (about 2.5 g), significantly exceeding the recommended values (1.5 g), while potassium intake remains moderate (2069.30 ± 761.67 mg) and slightly exceeds the recommended norms of 2000 mg. At the same time, calcium consumption (573.57 ± 181.72 mg) remains extremely low and amounts to only 44% of the recommended norm. Insufficient iron consumption is noted in this age group among girls (12.69 out of 20 mg) and amounts to 63.45% of the recommended norm and phosphorus (996.83 out of 1200 mg) amounts to 83.07% of the recommended norm. Magnesium levels correspond to or slightly deviate from the recommended values. With regard to vitamins, a decrease in vitamin C consumption (59.50 ± 55.25 mg) is noted compared to the younger group and amounts to 73.6% of the recommended norms for boys (75 mg) and 97.55% of the recommended norm for girls (65 mg), which may reflect a decrease in the proportion of fresh vegetables and fruits in the diet. Consumption of B vitamins remains at a moderate level, without significant deviations. Insufficient intake of vitamin A and E is noted, amounting to 559.36 ± 218.31 mcg of the recommended 800 mcg for vitamin A and 8.73 ± 2.76 mg of the recommended 15 mg for vitamin E (Table 3).

Depending on weight group, the highest protein intake was observed in obese adolescents (75.31 g of protein). As noted earlier, all weight categories were underfed in carbohydrates, with the highest intake observed in overweight adolescents (240.51 g). This age group also showed excess fat intake, with the highest intake observed in overweight (88.33 g) and obese (85.48 g).

In the older age group (15–17 years), the nutritional profile is characterized by the highest level of energy and macronutrient consumption compared to the younger groups, which corresponds to the physiological needs of adolescence. The average daily energy intake is 2460 kcal (sd = 557.90) and is generally almost 500 kcal less than the recommended norm for boys of 3000 kcal, while the daily energy intake of girls is normal. The diet of adolescents in this group, like younger children, is distinguished by an increased consumption of fats (100.21 ± 29.21 g), which exceeds the recommended norms, especially for girls (101 g out of 75 g recommended), with a simultaneous increase in the proportion of saturated fatty acids (40.02 ± 13.32 g). Carbohydrate intake (300.85 ± 76.71) still deviates from the recommended norms, especially in boys (297.51 g out of 450 g recommended for boys and 304 g out of 360 g recommended for girls), but a significant increase in the proportion of simple sugars is noted (165.52 ± 55.40). Protein intake (81.21 ± 21.72 g) also generally does not meet the recommended norms for boys (80.81 out of 90 g recommended for boys). Analysis of mineral intake shows that the sodium level remains significantly elevated (about 2.5 g), exceeding the recommended values (1.5 g), which is a stable unfavorable trend. Potassium intake (2941.61 ± 860.97 mg) also exceeds the recommended norms (2000 mg). Calcium intake (572.41 ± 192.60 mg) remains significantly below the recommended level (1300 mg). Similarly, magnesium and phosphorus intake are within or slightly above/below the recommended values. Particularly noteworthy is the iron intake level (17.02 ± 4.78 mg), which, on average, girls in this age group consume less of than the recommended value (17.47 out of 20 mg recommended). The vitamin profile is characterized by high intake of vitamin C (121.35 ± 88.35 mg), which exceeds the recommended norms by 61%. The trend in B vitamin intake (B1, B2, B3) is characterized by compliance with the recommended values. Vitamin E intake (11.37 ± 4.46 mg) is also below the norm (15 mg recommended) (Table 3).

An analysis of older adolescents’ nutrition by weight category showed that protein intake in all weight categories met the recommended daily intake of 72–90 g. The highest protein intake is observed in the overweight group of older adolescents (87.73 g). All weight categories demonstrate underconsumption of the recommended carbohydrate intake, with the highest carbohydrate intake observed in obese older adolescents (316.94 g). All weight categories demonstrate excessive fat intake, especially in the overweight and obese groups (100.74 and 100.82 g, respectively). Daily energy intake in all groups is slightly lower than the recommended intake (3000 kcal for boys and 2400 kcal for girls), ranging from 1982.00 calories in underweight children to 2513.61 calories in obese children.

**Figure 1:**
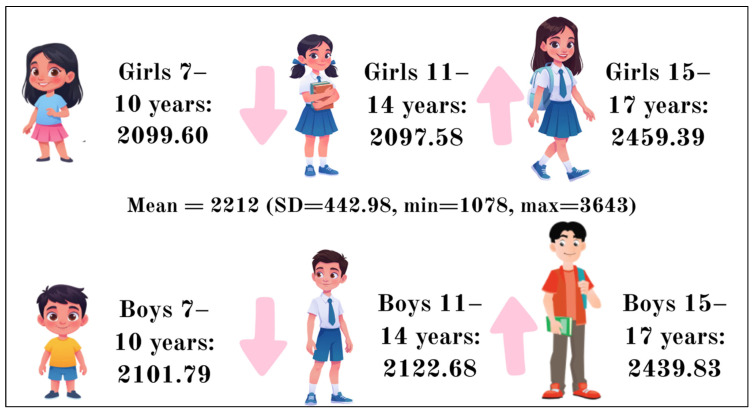
Daily calorie intake by gender and age group.

Figure 1 shows the average daily calorie intake of schoolchildren aged 7–17 years, broken down by gender and age group. Overall, the average daily calorie intake among all children was 2225.9 kcal, with a standard deviation of 472.34. The minimum recorded value was 721.1 kcal, and the maximum was 4344.9 kcal. Among girls aged 7–10 years, the average daily calorie intake was 2099.60 kcal. In the 11–14 age group, this figure decreased slightly to 2097.58 kcal. However, in the older age group (15–17 years), the average daily calorie intake increased significantly, reaching 2459.39 kcal. For boys aged 7–10, the average daily calorie intake was 2101.79 kcal, which is almost identical to that of girls of the same age. Among the 11–14-year-old group, there was a slight increase to 2122.68 kcal, and among the older 15–17-year-old group, the figure rose to 2439.83 kcal.

**Figure 2:**
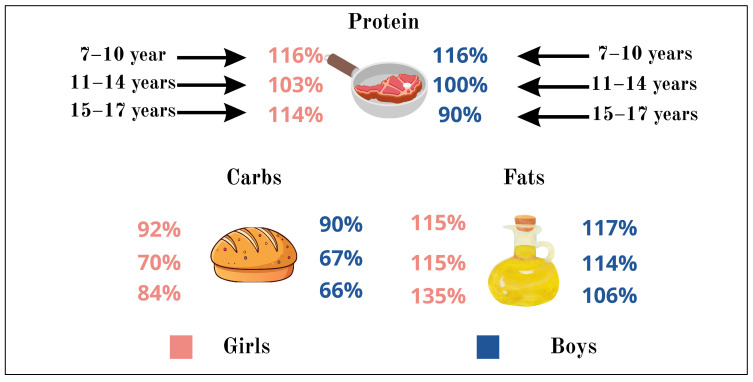
Protein, fat, and carbohydrate intake of the children studied, depending on gender and age.

Figure 2 shows the protein, fat, and carbohydrate intake of the children studied, depending on gender and age, compared with the recommended values of the National Center for Healthy Nutrition of the Republic of Kazakhstan. For all studied indicators, children in all age groups, both boys and girls, exceeded the recommended daily intake of protein, and fat. Carbohydrate intake was below the recommended values, especially for boys in the 15–17 age group (66% of the recommended daily intake). It was also noted that adolescent boys did not achieve the recommended daily intake of protein (90%).

### 3.3. Factors Associated with Nutritional Composition of Schoolchildren’s Diets

Factors associated with dietary intake included age, school shift, district of residence, family income, and food-related expenses (Table 4).

Older age was consistently associated with higher intake of several nutrients. Children aged 11–14 and 15–17 years had significantly higher liquid consumption compared to those aged 7–10 years (*p* < 0.001). Similarly, adolescents aged 15–17 years demonstrated higher daily calorie and protein intake (*p* < 0.001). Fat and saturated fatty acid intake were also significantly higher in both older age groups (*p* < 0.001). Cholesterol intake increased with age as well, with both age groups showing significantly higher values compared to younger children (*p* < 0.05).

At the same time, older age was associated with lower intake of certain nutrients. Children aged 11–14 years had lower mono- and disaccharide, carbohydrate, dietary fiber, and ash intake compared to those aged 7–10 years (*p* < 0.05), while adolescents aged 15–17 years had lower starch intake (*p* < 0.001).

School-related factors also played a role. Morning shift attendance was associated with lower liquid and cholesterol intake compared to the afternoon shift (*p* < 0.05). In addition, children studying in schools located in the “Maikuduk” district had lower intake of calories, mono- and disaccharides, carbohydrates, and dietary fiber compared to those from the “South-East” district (*p* < 0.05).

Socioeconomic factors were significantly associated with dietary patterns. Higher family income (>1200 USD) was associated with lower intake of calories, fats, mono- and disaccharides, and total carbohydrates (*p* < 0.05). In contrast, children with unknown family income had higher liquid intake (*p* < 0.05). Furthermore, children with unknown monthly food expenses had higher fat intake compared to those with lower food expenses (*p* < 0.05).

Ethnicity was also associated with dietary intake: children of Kazakh ethnicity had higher carbohydrate intake compared to non-Kazakh children (*p* = 0.046).

## 4. Discussion

Our data from Karaganda schoolchildren presented a coherent pattern: caloric intake rose from childhood through late adolescence at a rate consistent with developmental energy requirements [34,36,37], yet diet quality deteriorated in parallel. The additional energy consumed by older children and adolescents was derived primarily from fat and free sugars rather than from complex carbohydrates or protein from diverse dietary sources, which meant that caloric sufficiency and nutritional adequacy came apart across the age range studied. This is consistent with patterns reported in children’s populations in different parts of the world [27,35].

The macronutrient structure of the sample was consistent with what the literature describes as a Western-type dietary pattern in children and adolescents [38,39]. Fat intake increased progressively across age groups, and by late adolescence, fat energy as a share of total calories approached or exceeded the upper limit that the WHO/FAO joint expert consultation recommends [40]. Saturated fatty acid intake rose in parallel with total fat, and dietary cholesterol peaked in the 11–14 group at precisely the developmental stage when pubertal cardiovascular risk stratification begins. A study on the pathobiological determinants of atherosclerosis in youth documented that high dietary cholesterol and saturated fat during adolescence are associated with early atherosclerotic lesion formation and adverse lipid profiles by young adulthood [41], and the fat and cholesterol data for the 11–14 group described the dietary conditions that study identified as precursors to those outcomes.

Protein intake exceeded the national norms for most age–sex subgroups [34], though the surplus was qualitatively specific. The diet was heavily animal-sourced, which matters beyond the quantity. Early-life research implicates high animal protein intake in activation of the IGF-1 axis, connecting protein intake during growth with accelerated bone development, earlier puberty, and fat accumulation [42,43]. On a broader scale, a food frequency survey in five Kazakhstani regions found about one in four young adults consumed meat every day, and their weekly intake was nearly twice the World Cancer Research Fund’s recommended limit [44]. Jia and colleagues documented that per capita protein consumption in Kazakhstan more than doubled between 2001 and 2018, with the Karaganda region among the highest-contributing areas [21], and the present sample reflected the individual-level expression of that trend. Boys aged 15–17 were the one subgroup showing a protein shortfall, reaching approximately 90% of their sex-specific recommendation. This gap matters because pubertal males accumulate lean mass at rates that substantially elevate protein requirements, and deficits at this stage are not easily compensated later [45,46].

The carbohydrate fraction showed an internal compositional shift as children aged. Starch intake declined progressively while free sugar intake rose, reaching levels that, relative to total energy intake, substantially exceeded the WHO upper limit of 10% of daily energy from free sugars [47]. Older adolescents were not consuming more carbohydrates overall. They were consuming qualitatively inferior carbohydrates. This substitution of complex carbohydrates by simple sugars across adolescence is well-documented in the nutrition transition literature [48,49] and has been recorded specifically for Kazakhstan using food supply data [21]. Dietary fiber intake across the full sample remained well below EFSA [50] and Institute of Medicine [51] recommendations, with the deepest deficit in the 11–14 group. Reynolds and colleagues, drawing on systematic reviews and meta-analyses published in The Lancet, established dose–response relationships between fiber intake and reduced incidence of coronary heart disease, stroke, type 2 diabetes, and colorectal cancer that hold at the low absolute intake levels observed in this sample, not only at higher consumption [52]. Rising simple sugars in older adolescents and chronically low fiber across all ages describe the compositional profile that the literature associates with adverse cardiometabolic risk [53,54]. European multi-country pediatric data confirm that energy-dense, nutrient-poor dietary patterns carry systematically lower fiber provision [55], and ultra-processed food intake reliably produces this combination [56]. Russian school-age dietary data had a similar pattern [57].

Sodium intake showed almost no developmental variation across the age range studied. The three group means differed by less than 0.5%, and all three exceeded the WHO adult ceiling of 2000 mg/day [58] by approximately 25%. Potassium intake increased across age groups and exceeded WHO-recommended levels at any stage [59]. The resulting sodium-to-potassium ratio was unfavorable across all three age groups, with the most adverse values recorded in children aged 7–14. Because potassium attenuates the blood-pressure effect of sodium [60,61], the ratio between the two electrolytes is the more informative cardiovascular exposure metric, and a ratio that starts adversely in early childhood and does not improve through adolescence is associated with elevated cardiovascular risk in adulthood [61,62,63]. He and MacGregor’s meta-analysis of controlled trials demonstrated that even modest sodium reductions produce measurable blood pressure decrements in children and adolescents [60]. The near-absence of any developmental change in sodium intake across ten years of growth was most plausibly explained by sodium-dense foods constituting a fixed structural component of the dietary environment throughout the entire school-age period, rather than by a behavior that shifts with age.

The 11–14-year age group showed simultaneous shortfalls in calcium, iron, magnesium, vitamin C, vitamin E, and thiamin. This clustering was not coincidental and reflected a common dietary substrate. The food groups with the largest deficits in this age group were precisely those that supply these micronutrients most reliably: dairy products (calcium), whole grains (thiamin, magnesium, fiber), legumes and leafy vegetables (iron, magnesium, vitamin C), and nuts and seeds (vitamin E, magnesium). When these food groups are displaced by energy-dense alternatives, their micronutrient contributions decline together, not independently.

Calcium was the most clinically consequential deficit in the sample at this developmental stage. Approximately 40–45% of lifetime bone mineral mass is accreted during adolescence [64,65], and the gap between calcium supply and demand widened precisely when it should be closing. Bonjour and colleagues reported in a randomized trial that two years of calcium-enriched food provision in prepubertal girls produced measurable increases in bone mineral mass [66], establishing that dietary calcium shortfalls during growth have directly quantifiable skeletal consequences. Regional bone mineral density data from Kazakhstan document high rates of low bone density in children and adolescents [67], consistent with the calcium intakes observed in the present sample. The Kazakhstani context amplifies this risk further: vitamin D insufficiency affects approximately 56% of Kazakhstani children [68], impairing intestinal calcium absorption and deepening the functional deficit beyond what dietary intake data alone indicate.

Iron inadequacy at 11–14 years carried a sex-specific dimension that group means tend to obscure. The Kazakhstan national norm for girls in this age group reflects the additional iron requirement introduced by menarche, and sex-stratified analysis identified the largest iron gap in adolescent girls. The intake figure itself understated the functional nutritional risk, because non-heme iron, the predominant form in this population’s dietary pattern, is subject to absorption inhibitors structurally embedded in the diet. Polyphenols in black tea, a beverage consumed regularly in this population, reduce non-heme iron absorption by 60–90% relative to water, and phytates in refined wheat products impose an independent absorption penalty [69]. At precisely the stage of greatest iron demand, vitamin C intake fell below the Kazakhstan recommendation, removing the primary dietary enhancer of non-heme iron bioavailability. The convergence of low absolute intake, possible high inhibitor exposure, and low enhancer intake placed adolescent girls at substantially greater iron nutritional risk than the group mean conveyed. Iron deficiency and iron-deficiency anemia are documented public health problems in Kazakhstan, with higher anemia prevalence reported in girls compared with boys in studies of rural schoolchildren in the country [70].

Thiamin intake fell below of the normative reference in all age groups and the fiber and whole grain deficit could be tracked through the same underlying mechanism: refined cereals and added sugars displace the whole grain foods that supply thiamin. Because thiamin is an obligate cofactor in carbohydrate metabolism and neurological function [71], this represented a functionally relevant inadequacy at a developmental stage simultaneously showing the lowest energy intake relative to estimated requirements in the sample. Vitamin E intake was below the norm across all age groups, with the largest absolute gap in the 11–14 cohort. The non-monotonic relationship with age followed the fat intake trajectory, given that dietary vitamin E in this population is derived primarily from fat-containing foods.

Childhood vitamin E insufficiency is a relatively underrecognized issue in pediatric nutrition [72], and biomarker data would be needed to establish functional status. Magnesium showed a related deficit pattern. The same food groups absent from the diet, namely whole grains, legumes, nuts, and leafy greens, supply magnesium alongside fiber and B vitamins, so their collective under-consumption produces overlapping shortfalls rather than isolated ones.

Vitamin C intake followed a non-linear trajectory across the three age groups, declining from 83.2 mg/day at 7–10 years to 59.5 mg/day at 11–14 years before increasing to 121.4 mg/day at 15–17 years. This pattern tracked the behavioral dynamic documented in European cohort data [73,74], where fruit and vegetable consumption falls during early adolescence as children gain food autonomy and gravitate toward peer-influenced, energy-dense food choices before partially recovering later. The vitamin C deficit in the 11–14 group carried a secondary nutritional consequence already identified above. Dietary vitamin C is the primary enhancer of non-heme iron absorption [69], and its decline at precisely the age of highest iron demand in girls compounded the bioavailability problem. Vitamin A intake was below the Kazakhstan norm in the 11–14 group, adding another micronutrient gap to the cluster at this age. National dietary trend data indicate a declining share of vitamin A-rich foods in Kazakhstani children’s diets as energy-dense alternatives have expanded [21]. Serum retinol measurement would be required to characterize functional vitamin A status, as dietary recall data alone are insufficient to resolve this question.

The multivariate regression results revealed a socioeconomic pattern inconsistent with a linear poverty model. Children from middle-income households showed higher energy and fat intake than those from the highest-income group, and free sugar intake was elevated in the upper-middle income category relative to the highest income group. Children from the lowest-income households did not consistently show the most unfavorable dietary profiles across the outcomes examined. This pattern was interpretable within the nutrition transition framework described by Popkin [48,49]: at intermediate income levels, purchasing power expands access to energy-dense processed foods while health literacy, market access, and food environment conditions have not yet shifted consumption toward more nutrient-dense alternatives. Higher food spending did not reliably translate into more favorable outcomes across the dietary measures examined, a finding that reinforced the argument that food environment structure, not purchasing power alone, determines what children eat.

Paternal education emerged as a more informative household variable than maternal education in this sample, since maternal education was near-universally high and therefore unable to differentiate dietary outcomes. After adjustment for income, food expenditure, age, and shift, children whose fathers had secondary or vocational education showed higher dietary cholesterol than those whose fathers held university degrees. Afternoon school shift attendance was independently associated with higher dietary cholesterol compared with morning shift attendance after adjustment for age, income, and other covariates, consistent with evidence from Mexican adolescents showing that shift timing disrupts regular meal patterns and increases reliance on energy-dense ready-to-eat foods outside structured mealtimes [75]. Because younger children were more heavily represented in the afternoon shift and older students in the morning shift, age and shift effects were partially confounded in this sample, and their independent contributions to dietary outcomes could not be fully separated in a cross-sectional design. District-level variation in carbohydrate intake, with higher values in the “South-East” district after controlling for age, income, and shift, suggested a genuine local food environment contribution to within-city dietary differences. A mixed-methods study in Karaganda documented high exposure to digital food advertising for sugar-sweetened beverages, energy drinks, and fast food among schoolchildren in this setting [76].

Traditional Kazakhstani dietary practices, though diminishing in urban settings, may attenuate adiposity slightly at a population level [21], but sex- and ethnicity-stratified nutrient analyses would be needed to identify the specific dietary sources of this BMI difference. A principal component analysis of adult dietary patterns in Aktobe found a measurable generational shift toward a processed-food-dominated pattern in younger adults [77], suggesting that the age-related dietary trajectory observed in Karaganda schoolchildren was part of a broader population-level change rather than a cohort-specific phenomenon.

Individual-level dietary assessment data collected in a defined urban population of this size and demographic specificity are rare for Central Asia. Existing estimates for Kazakhstan have relied primarily on national food supply data [21] or broad regional surveys, both of which overestimate average individual intake and cannot capture within-population dietary heterogeneity. The present data characterized a specific urban setting. Karaganda is Kazakhstan’s second-largest city, with pronounced ethnic heterogeneity, a shift schooling system producing structured variation in meal timing, a documented commercial food marketing environment directed at children [76], and a per capita dairy intake trajectory that has declined over the past two decades [21]. These features made it a contextually distinct setting whose findings complemented rather than duplicated what is known from the regional literature. A review of school-age nutrition across Europe and Central Asia identified high sodium and sugar intake, insufficient calcium, low fiber, and inadequate fruit and vegetable consumption as common features of post-Soviet and Central Asian dietary environments [78]. The present data confirmed these patterns at the individual level in a Kazakhstani urban population and added a socioeconomic gradient analysis not available from food supply data.

The revised national school nutrition standard, which entered force in September 2025 and mandates reductions in sugar of up to 3.6-fold and in salt of up to fivefold, alongside increased provision of vegetables, fruits, and dairy products [79], directly addresses the deficits documented in this study. The present dataset, collected immediately before implementation, constitutes an empirical baseline against which compliance effects can be evaluated in future surveillance rounds. Effective implementation will require supply chain development for dairy and fresh produce, training of food service personnel, and monitoring structured at the district level rather than aggregated city-wide. The finding that middle-income families, not the lowest-income group, tended to generate the most energy-dense and highest-fat dietary patterns means that intervention strategies calibrated around household poverty will bypass a substantial share of the population with unfavorable dietary profiles. Restrictions on food marketing directed at children, product reformulation requirements, and food environment regulation near schools represent structural measures that nutrition education delivered through schools alone cannot substitute for, and they are warranted by the evidence from this setting.

This study has several methodological constraints that bear on the interpretation of its findings. The primary analytical constraint is the single 24 h recall design. One recall day cannot characterize habitual intake or adjust for intra-individual day-to-day variation, and this limitation produces substantial random classification error when individual adequacy is the target of inference [80]. Social desirability bias and recall error impact every self-reported dietary assessment, and for children between 7 and 10, parental recall adds another layer of uncertainty, especially when teachers estimate school meal portion sizes. Food group composition, ultra-processed food exposure, physical activity, meal timing, and pubertal stage were not measured directly, so there is only so much we can say about the dietary patterns we observed. Group means describe population trends but cannot quantify the prevalence of nutrient inadequacy at the individual level.

## 5. Conclusions

A study showed that as schoolchildren in Karaganda age, there is a clear increase in dietary calorie intake, but this is accompanied by a deterioration in dietary quality: the increase in energy comes primarily from fats and simple sugars, with inadequate intake of dietary fiber, complex carbohydrates, and several key micronutrients. The most pronounced nutritional imbalances were found in the 11–14-year-old group, where deficiencies in calcium, iron, magnesium, vitamins, and fiber are observed alongside high consumption of sodium, fats, and sugars, resulting in an unfavorable cardiometabolic profile.

Socioeconomic factors, including income level, food expenses, father’s education, and the organization of the educational process (shift schooling), significantly influence dietary patterns, with more unfavorable diets more often found in middle-income families than in low-income families.

The findings highlight the need for comprehensive interventions beyond school meals, including regulating the food environment, reducing the availability of ultra-processed foods, increasing the availability of dairy products, fruits and vegetables, and developing systems for monitoring and evaluating the effectiveness of the updated school meal standards in Kazakhstan.

## Figures and Tables

**Table 1:** Sample description.

Variable	Total, *n* = 865 (%)
Male/female	414 (47.86)/451 (52.14)
Children of Kazakh ethnicity/non-Kazakh ethnicity	478 (55.26)/387 (44.74)
School in “Maikuduk” district/“South-East” district	399 (46.13)/466 (53.87)
Morning shift/afternoon shift	619 (71.56)/246 (28.44)
Family income less than 950$/950–1200$/more than 1200$/unknowm	282 (32.60)/149 (17.23)/193 (22.31)/241 (27.86)
Monthly food expenses less than 310$/310–430$/more 430$/unknown	278 (32.14)/278 (32.14)/264 (30.52)/45 (5.20)
Mother’s education—secondary school/higher	190 (21.97)/675 (78.03)
Father’s education—secondary school/higher	269 (31.09)/510 (58.96)

**Table 2:** Distribution of BMI-for-age categories by age group.

	Total		7–10 Years				11–14 Years				15–17 Years			
			Girls (*n* = 155)		Boys (*n* = 133)		Girls (*n* = 162)		Boys (*n* = 147)		Girls (*n* = 134)		Boys (*n* = 134)	
	*n*	%	*n*	%	*n*	%	*n*	%	*n*	%	*n*	%	*n*	%
Underweight	6	0.70	1	0.65	1	0.75	0	0	0	0	0	0	4	2.98
Normal	728	84.16	131	84.52	113	84.96	131	80.86	125	85.03	111	82.83	117	87.31
Overweight	81	9.36	14	9.03	7	5.26	25	15.43	14	9.52	16	11.94	5	3.73
Obesity	50	5.78	9	5.80	12	9.03	6	3.71	8	5.45	7	5.22	8	5.97

**Table 3:** Summary statistics of actual dietary intake of students aged 7–17 in general education schools of Karaganda per day.

Substance	Mean ± SD (Min/Max)	Children Age Group, Mean ± SD (Min/Max)		
		7–10 Years	11–14 Years	15–17 Years
Liquid (mL)	1751 ± 312.14 (1064/2429)	1418.28 ± 187.33 (1064.03/1973.67)	1755.47 ± 120.50 (1405.66/2129.46)	2102.28 ± 132.36 (1718.82/2429.21)
Proteins (g)	76.16 ± 18.61 (28.17/150.60)	72.99 ± 14.64 (35.48/127.35)	74.73 ± 18.14 (28.17/150.60)	81.21 ± 21.72 (28.96/142.96)
Fats (g)	83.12 ± 27.32 (19.18/189.35)	75.14 ± 20.43 (19.18/161.92)	87.79 ± 26.09 (20.02/173.39)	100.21 ± 29.21 (25.14/189.35)
Carbs (g)	278.63 ± 71.63 (91.19/663.26)	287.02 ± 64.32 (120.38/663.26)	251.53 ± 64.84 (91.19/449.08)	300.85 ± 76.71 (94.74/563.435)
SFA (g)	36.64 ± 12.30 (9.27/86.38)	33.12 ± 9.90 (9.27/71.74)	36.99 ± 12.56 (9.42/75.65)	40.02 ± 13.32 (10.69/86.38)
Cholesterol (mg)	431.7 ± 274.21 (46.4/1695.5)	402.02 ± 245.04 (75.6/1695.55)	470.18 ± 304.98 (50/1653.80)	419.29 ± 262.03 (46.4/1178.90)
Mono/disaccharides (g)	131.31 ± 58.24 (30.04/363.87)	124.02 ± 40.69 (54.96/262.84)	108.44 ± 60.97 (30.04/293.12)	165.52 ± 55.40 (53.63/363.87)
Starch (g)	137.22 ± 53.15 (11.51/455.98)	161.74 ± 60.48 (41.46/455.98)	130.09 ± 44.07 (29.96/278.10)	119.09 ± 43.96 (11.51/246.16)
Dietary fiber (g)	15.70 ± 5.23 (5.07/34.17)	17.32 ± 4.62 (6.26/34.08)	13.00 ± 4.29 (5.72/26.51)	17.08 ± 5.57 (5.07/34.17)
Organic acids (g)	6.07 ± 4.18 (0.57/28.06)	4.23 ± 2.11 (1/9.74)	4.57 ± 2.35 (0.57/15.20)	9.77 ± 5.05 (1.54/28.06)
Ash (g)	16.45 ± 4.44 (5.48/37.57)	16.36 ± 3.99 (8.97/34.37)	15.39 ± 3.59 (8.05/28.06)	17.76 ± 5.35 (5.48/37.57)
Na (mg)	2522 ± 690.11 (1074/3886)	2515.63 ± 791.46 (1083.1/3885.5)	2523.98 ± 606.15 (1078.9/3514)	2526.77 ± 666.25 (1073.5/3743.1)
K (mg)	2311.6 ± 858.88 (780.8/6045.9)	1985.35 ± 606.53 (799.3/4023.15)	2069.30 ± 761.67 (780.8/5138.25)	2941.61 ± 860.97 (1074.5/6045.90)
Ca (mg)	577.9 ± 185.07 (300.6/1204.2)	587.59 ± 181.71 (359.79/999.4)	573.57 ± 181.72 (318.00/1115.7)	572.41 ± 192.60 (300.55/1204.2)
Mg (mg)	254.9 ± 81.91 (95.4/607.8)	232.62 ± 65.75 (100.6/446.45)	228.49 ± 63.85 (95.4/409.80)	309.39 ± 89.65 (100.2/607.80)
P (mg)	1025.4 ± 261.63 (320.4/2217.3)	966.42 ± 200.74 (544.7/1824.25)	996.83 ± 236.22 (554.6/1739.40)	1121.75 ± 315.93 (320.4/2217.30)
Fe (mg)	14.94 ± 4.26 (5.80/30.67)	15.26 ± 3.53 (5.99/27.43)	12.85 ± 3.37 (6.24/22.74)	17.02 ± 4.78 (5.80/30.67)
Vitamin A (mcg)	670.4 ± 330.90 (276.8/2803.9)	559.36 ± 218.31 (276.8/993.6)	672.26 ± 257.49 (300.6/1420.7)	787.46 ± 446.72 (301.5/2803.9)
Vitamin E (mg)	9.46 ± 3.69 (2.03/29.83)	8.73 ± 2.76 (4.03/22.08)	8.49 ± 3.07 (3.20/19.80)	11.37 ± 4.46 (2.03/29.83)
Vitamin B1 (mg)	0.87 ± 0.29 (0.29/2.52)	0.79 ± 0.21 (0.30/1.62)	0.82 ± 0.26 (0.39/1.72)	1.01 ± 0.36 (0.29/2.52)
Vitamin B2 (mg)	1.12 ± 0.41 (0.33/3.57)	0.97 ± 0.30 (0.41/2.38)	1.15 ± 0.45 (0.33/3.57)	1.23 ± 0.42 (0.44/3.13)
Vitamin B3 (mg)	14.58 ± 6.09 (0.68/58.11)	12.86 ± 3.71 (6.07/34.63)	13.18 ± 5.87 (4.40/41.94)	18.04 ± 6.95 (0.68/58.11)
Vitamin C (mg)	86.55 ± 73.24 (0.56/589.36)	83.18 ± 60.66 (5.20/422.62)	59.50 ± 55.25 (0.56/287.69)	121.35 ± 88.35 (16.92/589.36)
Niacin equivalent (mg)	28.07 ± 9.11 (0.87/76.84)	27.54 ± 6.48 (15.90/60.39)	25.86 ± 7.80 (10.24/59.18)	31.19 ± 11.74 (0.87/76.84)

**Table 4:** Multivariable regression analyses of factors associated with nutritional composition of schoolchildren’s diets.

Categories	B	95% CI		*p*-Value
Liquid				
aged 11–14 years (ref.: aged 7–10)	345.649	319.402	371.896	<0.001
aged 15–17 (ref.: aged 7–10)	694.501	665.536	723.465	<0.001
morning shift (ref.: afternoon shift)	−31.194	−56.930	−5.457	0.018
unknown family income (ref.: family income less than 950)	30.631	3.419	57.842	0.027
Daily calorie intake				
school in “Maikuduk” district (ref.: school in “South-East” district)		−128.715	−6.934	0.029
aged 15–17 (ref.: aged 7–10)	319.404	232.672	406.136	<0.001
family income more than 1200$ (ref.: family income less than 950)	−109.065	−195.163	−22.967	0.013
Proteins				
aged 15–17 (ref.: aged 7–10)	8.157	4.573	11.740	<0.001
Fats				
aged 11–14 years (ref.: aged 7–10)	13.734	9.239	18.229	<0.001
aged 15–17 (ref.: aged 7–10)	26.244	21.284	31.204	<0.001
family income more than 1200$ (ref.: family income less than 950)	−4.987	−9.911	−0.063	0.047
unknown monthly food expenses (ref.: monthly food expenses less than 310$)	8.706	0.396	17.016	0.040
SFA				
aged 11–14 years (ref.: aged 7–10)	4.657	2.540	6.774	<0.001
aged 15–17 (ref.: aged 7–10)	7.922	5.586	10.258	<0.001
Cholesterol				
aged 11–14 years (ref.: aged 7–10)	95.581	47.697	143.464	<0.001
aged 15–17 (ref.: aged 7–10)	61.639	8.799	114.480	0.022
morning shift (ref.: afternoon shift)	−76.764	−123.716	−29.813	0.001
Mono/disaccharides				
school in “Maikuduk” district (ref.: school in “South-East” district)	−7.562	−14.818	−0.305	0.041
aged 11–14 years (ref.: aged 7–10)	−15.828	−25.195	−6.461	0.001
aged 15–17 (ref.: aged 7–10)	41.597	31.260	51.933	<0.001
family income more than 1200$ (ref.: family income less than 950)	−12.726	−22.987	−2.465	0.015
Starch				
aged 11–14 years (ref.: aged 7–10)	−34.002	−42.873	−25.132	<0.001
aged 15–17 (ref.: aged 7–10)	−44.744	−54.533	−34.955	<0.001
Carbs				
school in “Maikuduk” district (ref.: school in “South-East” district)	−14.178	−23.476	−4.880	0.003
aged 11–14 years (ref.: aged 7–10)	−40.050	−52.052	−28.049	<0.001
children of Kazakh ethnicity (ref.: children of non-Kazakh ethnicity)	9.340	0.175	18.504	0.046
family income more than 1200$ (ref.: family income less than 950)	−15.816	−28.963	−2.669	0.018
Dietary fiber				
school in “Maikuduk” district (ref.: school in “South-East” district)	−1.001	−1.659	−0.343	0.003
aged 11–14 years (ref.: aged 7–10)	−4.615	−5.464	−3.765	<0.001
Ash				
aged 11–14 years (ref.: aged 7–10)	−0.857	−1.624	−0.091	0.028
aged 15–17 (ref.: aged 7–10)	1.540	0.695	2.386	<0.001
VIF < 3				

## Data Availability

The data presented in this study are available on reasonable request from the corresponding author due to privacy and ethical reasons.
